# Age influence on resistance and deformation of the human sutured meniscal horn in the immediate postoperative period

**DOI:** 10.3389/fbioe.2023.1249982

**Published:** 2024-01-05

**Authors:** Alejandro Peña-Trabalon, Ana Perez-Blanca, Salvador Moreno-Vegas, M. Belen Estebanez Campos, Maria Prado-Novoa

**Affiliations:** Clinical Biomechanics Laboratory of Andalusia (BIOCLINA), University of Malaga, Málaga, Spain

**Keywords:** human meniscal tissue, meniscal root detachment, suture, age influence, tissue resistance

## Abstract

**Introduction:** To preserve knee function, surgical repair is indicated when a meniscal root disinsertion occurs. However, this surgery has not yet achieved complete recovery of the joint´s natural biomechanics, with the meniscus-suture interface identified as a potentially determining factor. Knowing the deformation and resistance behavior of the sutured meniscal horn and whether these properties are preserved as the patient ages could greatly contribute to improving repair outcomes.

**Methods:** A cadaveric experimental study was conducted on human sutured menisci classified into three *n* = 22 age groups (young ≤55; 55 < middle-aged ≤75; 75 < old) were subjected to load-to-failure test by suture pulling. Meniscal thickness at the suture hole was measured and the applied traction force and tissue deformation in the suture area in the direction of traction were recorded during the test. The traction load that initiated the meniscal cut-out, $F_{c}$ , maximum load borne by the meniscus, $F_{u}$ , tissue stress at the cut-out initiation, $S_{c}$ , and equivalent stiffness modulus at the suture area, $m_{s}$ , were calculated.

**Results:** At the tissue level, the resistance in terms of $S_{c}$ decrease with age (young: 47.2 MPa; middle-aged: 44.7 MPa; old: 33.8 MPa) being significantly different between the young and the old group (*p* = 0.015). Mean meniscal thickness increased with age (young: 2.50 mm; middle-aged: 2.92 mm; old: 3.38 mm; *p* = 0.001). Probably due to thickening, no differences in resistance were found at the specimen level, i.e., in $F_{c}$ (overall mean 58.2 N) and $F_{u}$ (overall mean 73.6 N). As for elasticity, $m_{s}$ was lower in the old group than in the young group (57.5 MPa vs. 113.6 MPa, *p* = 0.02) and the middle-aged one (57.5 MPa vs. 108.0 MPa, *p* = 0.04).

**Conclusion:** Regarding the influence of age on the sutured meniscal horn tissue, *in vitro* experimentation revealed that meniscal horn specimens older than 75 years old had a more elastic tissue which was less resistant to cut-out than younger menisci at the suture hole area. However, a thickening of the meniscal horns with age, which was also found, leveled out the difference in the force that initiated the tear, as well as in the maximum force borne by the meniscus in the load-to-failure test.

## 1 Introduction

The anterior and posterior meniscal horns are attached to the subchondral bone of the proximal tibial surface by the meniscal roots, composed of longitudinal fibers that continue the circumferential fibers of the meniscal horn in their direct insertion into the tibial (Villegas et al., 2008; Wang et al., 2009; Johannsen et al., 2012). Meniscal roots are heavily involved in preserving the union between the meniscus and the bone, preventing its extrusion while allowing the meniscus sufficient mobility to adapt to the tibiofemoral contact area during knee flexion. Several biomechanical studies have found that detachment of the meniscal roots modify knee joint kinematics and contact biomechanics leading to alterations similar to meniscectomy (Ode et al., 2012; Perez-Blanca et al., 2016). Clinically, they lead to early cartilage loss, accelerating the development of pathologies such as arthritis and osteoarthritis (Jones et al., 2006).

As a result of the aforementioned studies, surgical treatment of meniscal root detachment has changed from partial meniscectomy to meniscal root reinsertion. The surgical repair is currently performed through two techniques: transtibial (Kim et al., 2006; Petersen and Zantop, 2006; Ahn et al., 2007; Feucht et al., 2013; Cerminara et al., 2014) and *in situ* fixation (Zantop et al., 2004; Kopf et al., 2011; Padalecki et al., 2014; Petersen et al., 2014; Cuéllar et al., 2017; Balke et al., 2018; Espejo-Reina et al., 2022). Both approaches hold the meniscus in place using suture threads that pass through and tract the tissue of the meniscal horn. Although these surgical procedures are common in clinical practice and have demonstrated partial recovery of the knee’s natural biomechanics, complete restoration has not yet been achieved (Lee et al., 2009; Moon et al., 2010; Seo et al., 2010; Kim et al., 2011; Espejo-Reina et al., 2022; Espejo-Reina et al., 2023)

Previous biomechanical work pointed to the tissue-suture interface as a factor of potential importance in improving root repair outcomes (Cerminara et al., 2014; Steineman et al., 2020). In this context, a better knowledge of the mechanical properties of the meniscal horn tissue in the area affected by the interaction with the suture could contribute to improving the surgical technique. It would also be of great interest to evaluate the age-dependence behavior of the meniscal tissue pulled by the suture, as it may be a limiting factor for the indication of surgery. Different experimental investigations have studied the mechanical properties of human meniscal tissue under various testing conditions, such as the uniaxial tensile test (Yan et al., 2016) or compression test at different physiological strain rates (Chia and Hull, 2008), but always dealing with the unaltered meniscal body and its attachments. However, to our knowledge, no published studies have addressed the mechanical behavior of human meniscal tissue of different age groups perforated and loaded by the suture thread.

This work has been designed to characterize the mechanical behavior of the meniscal horn tissue in the area affected by the passing suture in human specimens of different age groups in the early post-operative period, before the healing process occurs. The main hypothesis is that deformation and mechanical resistance of the meniscal horn tissue at the suture-interface surroundings change with age.

## 2 Materials and methods

After approval by the Ethical Committee of Experimentation of the University of Malaga, cryopreserved human knees obtained from adult donors from the mid-femur to the mid-tibia provided by a specialized company were used for the study, complying with all legal and ethical requirements.

### 2.1 Specimen preparation

To focus the study on the tissue-suture interface behavior, isolated cadaveric meniscus-suture constructs were tested. Knees were divided into three age groups: young, age ≤55; middle-aged, 55 < age ≤75; old, 75< age. They were stored individually frozen at −20°C in sealed plastic bags. One day before testing, a knee was brought to room temperature and wrapped in dampened gauze. Once thawed, it underwent dissection and both the medial and lateral menisci were carefully extracted, with special attention to preserving the horns and roots. Menisci were visually inspected to confirm their integrity and the absence of any pathologies. The inclusion criterion for a meniscus was to have a macroscopic quality grade of 3 or higher in according to the scale of Pauli et al., 2011. If the meniscus met this criterion, it was randomly assigned for either anterior or posterior horn repair. Subsequently, it was wrapped in a dampened gauze, placed in a sealed plastic bag and stored in the cooler until the testing phase. The inclusion criterion was satisfied by 66 menisci, which were then categorized into the three abovementioned age groups of N = 22.

**FIGURE 1:**
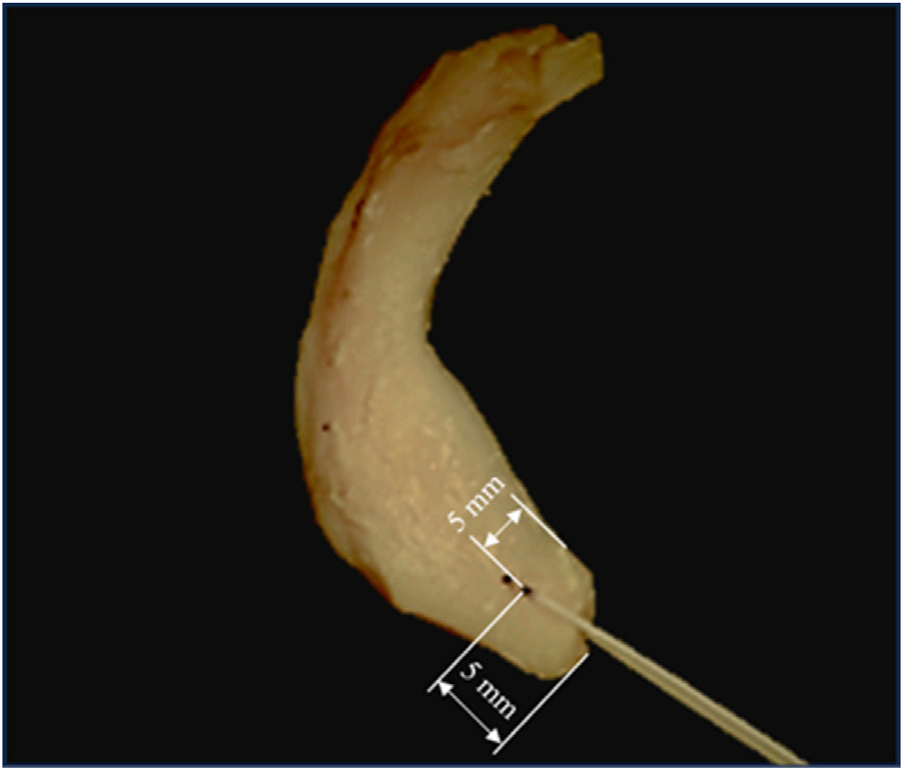
Sutured meniscus with N°2 non-absorbable, high resistance, 100% UHMWPE, braided fiber thread at 5 mm from its internal edge and its root junction. Marks are shown in an intact specimen that was discarded for not meeting the inclusion criterion.

At the time of testing, a N°2 non-absorbable, high-resistance, 100% UHMWPE, braided fiber thread (Force Fiber^®^ No. 2, Stryker Iberia, Madrid, Spain) was inserted by a specialized surgeon in the meniscal horn at 5 mm from both its internal edge and its root junction (Figure 1), using the attached ½ circle tapered needle. The puncture point was selected to match the zone where the surgical hole is normally made during the surgical procedure (Kim et al., 2006; Moon et al., 2010). The thickness of the meniscus at the insertion point was measured with a manual caliper.

To evaluate whether the influence of age was similarly preponderant in the lateral or medial site, the three age groups were divided into two subgroups of N = 11 specimens based on location.

### 2.2 Biomechanical testing

**FIGURE 2:**
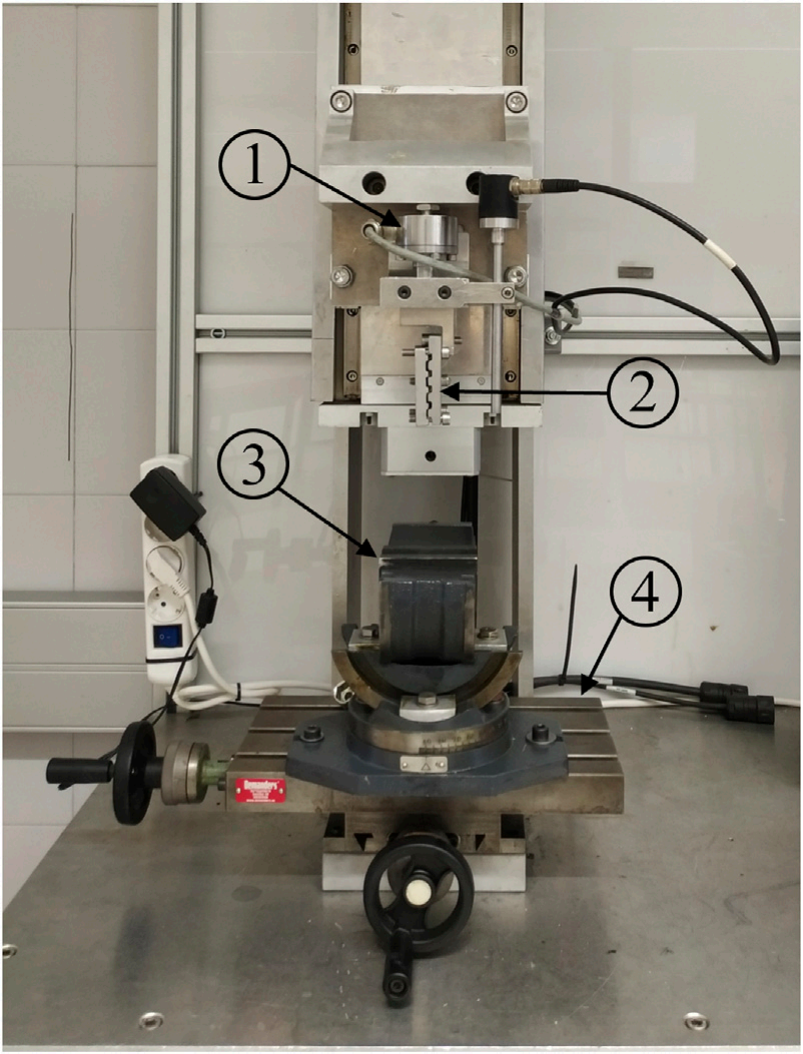
Uniaxial traction/compression testing machine: 1) load cell; 2) mechanical clamp to secure the suture free ends; 3) clamp with three orthogonal rotational degrees of freedom; 4) linear positioning table with two orthogonal linear degrees of freedom.

**FIGURE 3:**
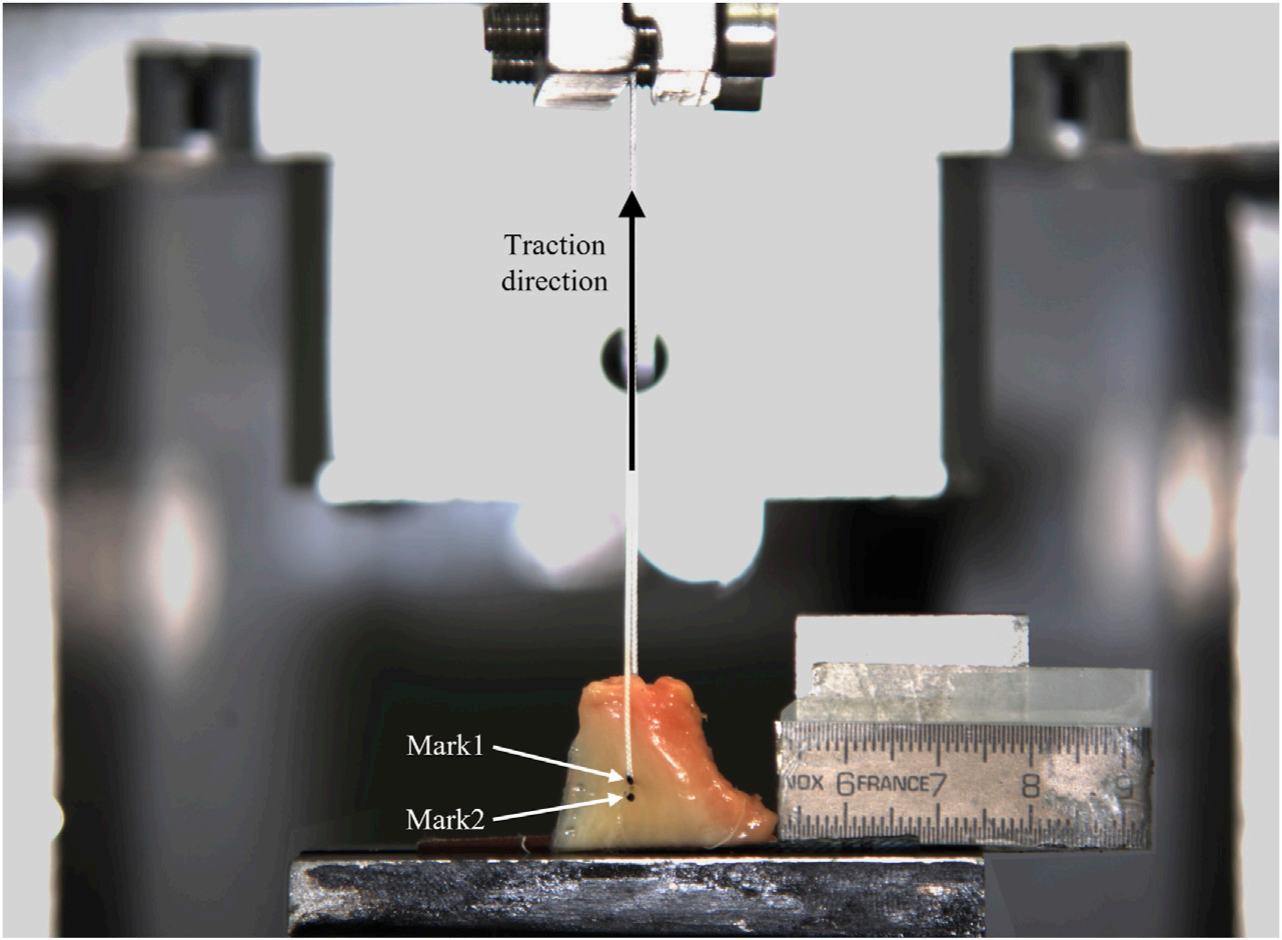
Experimental setup. Mark1 and Mark2 on the surface of one meniscus specimen can be observed.

A custom uniaxial testing machine of one column (Pérez de la Blanca, 2019) was used for the tests (Figure 2). Traction force was measured by a 2000 N load cell of accuracy class 0.1 (U2B, HBM, Darmstadt, Germany) and the actuator displacement by the servo-controller of the testing machine (SGDH-15AE-S-OY, Yaskawa Electric, Japan). Both signals sampled at 1,000 Hz. The meniscus was wrapped in gauze to keep it hydrated. To fix the specimen to the base of the testing machine, the tissue was placed between strips of sandpaper to increase friction and clamped at about 8 mm from the suture insertion point using a 5-degree-of-freedom clamp (TLT/SP-75, Wilton Tools, La Vergne, TN, United States). It was vertically positioned with the cranial surface facing the outer side of the testing machine. The longitudinal fibers of the horn were parallel to the loading direction and the suture hole situated in a pull line passing through the center of the machine actuator head. The two free ends of the suture were wrapped in sandpaper and secured to the machine head with a mechanical clamp. As a representative magnitude of the expected suture length in a transtibial repair (Cerminara et al., 2014; Perez-Blanca et al., 2018), 55 mm was left between the puncture point at the meniscus and the limit of the clamp when manually pulling the suture with only enough traction to keep it vertical. Once the specimen was placed in the testing machine, to check for possible slippage, lines were marked with a surgical pen on the meniscus and on the suture at the respective boundaries with the jaw and the clamp. Additionally, for videogrammetric analysis, two dots were marked on the horn aligned with the pulling direction of the thread (Figure 3). Marks were manually created using the tip of a surgical pen, resulting in an approximately circular shape which was subsequently measured. Mark1 was made on the suture limb at the cranial side of the meniscus and placed coincident with the meniscus-suture interface. Mark2 was made on the same meniscal surface and placed at the opposite side of the hole in the loading direction, as close to the hole as possible, but making sure that the ink did not contact its edges. The dots were positioned with the aim to analyze the relative elongation experienced by the tissue around the hole in the direction of traction.

A digital camera (VCXU-124C, Baumer, Switzerland) was placed in front of the testing machine, facing the meniscus cranial surface, carefully positioned with the image plane parallel to the medial transversal plane of the meniscus. The camera featured a CMOS sensor with a 4096 × 3000 pixels resolution and a 2.8/75 mm lens (C7528-M, Pentax, Japan). Images were recorded at a 250 ms sampling period and a 50 ms exposure time. An internal trigger was used to synchronize the recorded images to the sensors of the testing machine. Before the test started, a calibration image was taken using a measurement pattern.

The meniscus-suture construct was initially pulled up to 1 N at 0.05 mm/s, kept at this position for 5 s for stabilization and subsequently subjected to a traction load-to-failure test at 0.1 mm/s up to a maximum stroke of 20 mm.

### 2.3 Load-to-failure analysis

A videogrammetric software was developed in MATLAB^®^ to automatically identify the two marks, obtain their coordinates in each calibrated frame and compute the distance between them.

The evolution of the distance between the marks, D , was represented as a function of the traction force, *F*. From the curve, the instant of meniscal tissue cut-out was identified as the point of change in the slope (Pérez de la Blanca, 2019) and the force at this testing time identified as the meniscal cut-out force, $F_{c}$ . Additionally, the accuracy in detecting the cut-out initiation was verified by checking the images recorded during the test.

Also, the ultimate force, $F_{u}$ , i.e., the maximum force borne by the specimen in the load-to-failure test, was registered to allow the results of this work to be compared with other similar studies, since this is the most commonly reported variable related to meniscus resistance (Mitchell et al., 2016; Vertullo et al., 2021).

To characterize the meniscal tissue properties, tissue strain in the traction direction at the hole area was computed at each frame of the test as:

$$\varepsilon =\frac{D-D_{0}}{D_{0}}$$ (1)

where $D_{0}$ is the distance between the marks at the start of the load-to-failure test, computed for each specimen by the videogrammetric system on the first frame of the test, and D the distance between them at that frame. Tissue engineering stress in the traction direction at the hole was computed at each frame of the test as:

$$\sigma =\frac{F}{d_{t}∙t}$$ (2)

where F is the force recorded by the load cell at that frame. For the computation of σ , the fraction denominator is the projected suture-tissue contact area at the hole, $d_{t}$ is the thread diameter and t is the initial horn thickness measured at the hole. The tissue cut-out resistance, Sc, of the meniscal horn was calculated as the stress given by (2) at the tearing initiation as:

$$S_{c}=\frac{F_{C}}{d_{t}∙t}$$ (3)

Finally, equivalent stiffness modulus on the traction direction of the meniscal tissue area of the suture hole, $m_{s}$ , was computed as the slope of the lineal approximation of the stress-strain curve in the range of $\varepsilon =\left[0-0.3\right]$ .

### 2.4 Statistical analyses

To assess the influence of age on the mechanical properties of the human sutured meniscal horn, differences between the three age groups were evaluated for F_u_, F_c_, S_c,_ and m_s_. The sample size was selected based on S_c_ data from the first five specimens of each group (Mean: 42.2 MPa for young; 38.6 MPa for middle-age; 31.5 MPa for old and maximum group standard deviation (SD) of 10.5 MPa). Using G*Power 3.1.9.2 software (Faul et al., 2007) a large effect size (f = 0.44) for a one-way ANOVA test was estimated at this midpoint. A group size of *n* = 20 was found to be enough to detect a reduced effect size of 0.42, at α = 0.05 with a power of (1-β) = 0.80. Thus, accounting for a dropout rate of 0.1, a conservative group size of *n* = 22 was selected.

All other statistical analyses were performed using SPSS Statistics (v.20, IBM Corp). Percentage differences between group means were calculated with respect to the oldest group.

Overall differences for the three age groups in each mechanical property were assessed by conducting a one-way ANOVA test at α = 0.05. When a global significant difference was detected, between the groups, comparisons were carried out using Student’s t-tests with Bonferroni correction. With the chosen group size (*n* = 22), in the pairwise comparisons, a minimum detectable difference in *S*_*c*_ of 10 MPa (25% of resistance for the young group) was computed from the midpoint data.

As a secondary study, the influence of age was analyzed independently on the medial and lateral sides, splitting the samples in each age group according to location. Due to reduced group size resulting for this study, all data were tested for normality using a Shapiro-Wilk test and, as the data were not normally distributed, a non-parametric Kruskal-Wallis analysis of variance by ranks was chosen to evaluate statistical differences between age groups. If a global significant difference was found, pairwise comparisons were conducted with Mann-Whitney U tests with Bonferroni adjustment for multiple comparisons.

## 3 Results

The meniscus specimens were classified based on age, as outlined in the preceding section, yielding three groups characterized as follows:

• Young group: 11 medial and 11 lateral menisci, 11 anterior and 11 posterior horns, 18 men and 4 women, age 37.87, SD 6.25, median 41 years, range 28–47 years. Menisci were obtained from 12 cadaveric knees of 12 adult donors, 1 meniscus was discarded because it did not meet the inclusion criteria and another one in order not to exceed the N = 22 specimens in the group.
• Middle-aged group: 11 medial and 11 lateral menisci, 11 anterior and 11 posterior horns 8 men and 14 women, age 62.96, SD 3.97, median 64 years, range 57–67 years. Menisci were obtained from 12 cadaveric knees of 12 adult donors, 2 menisci were discarded because they did not meet the inclusion criteria.
• Old group: 11 medial and 11 lateral menisci, 10 anterior and 12 posterior horns, 10 men and 12 women, age 85.63, SD 4.51, median 83 years, range 82–95 years. Menisci were obtained from 14 cadaveric knees of 14 adult donors, 6 menisci were discarded because they did not meet the inclusion criteria.

Regarding the subgroups resulting from splitting each group into lateral and medial meniscus, they exhibited the following characteristics:

• For the young group: medial site with 4 anterior and 7 posterior horns, 9 men and 2 women, age 38.90, SD 6.15, median 41 years, range 28–47 years; and lateral site with 7 anterior and 4 posterior horns, 9 men and 2 women, age 37.83, SD 6.39, median 39 years, range 28–47 years.
• For the middle-aged group: medial site with 5 anterior and 6 posterior horns, 4 men and 7 women, age 61.89, SD 4.37, median 60 years, range 57–67 years; and lateral site with 6 anterior and 5 posterior horns, 4 men and 7 women, age 63.36, SD 4.13, median 64 years, range 57–67 years.
• For the old group: medial site with 5 anterior and 6 posterior horns, 5 men and 6 women, age 84.36, SD 3.26, median 83 years, range 82–91 years; and lateral site with 5 anterior and 6 posterior horns, 5 men and 6 women, age 85.40, SD 4.50, median 83 years, range 82–95 years.

The marks on the meniscal surfaces resulted inscribed in circles of mean radius of 0.40 mm (SD 0.07) ranging from 0.25 to 0.48.

### 3.1 Meniscal thickness at the suture-tissue interface, t

**FIGURE 4:**
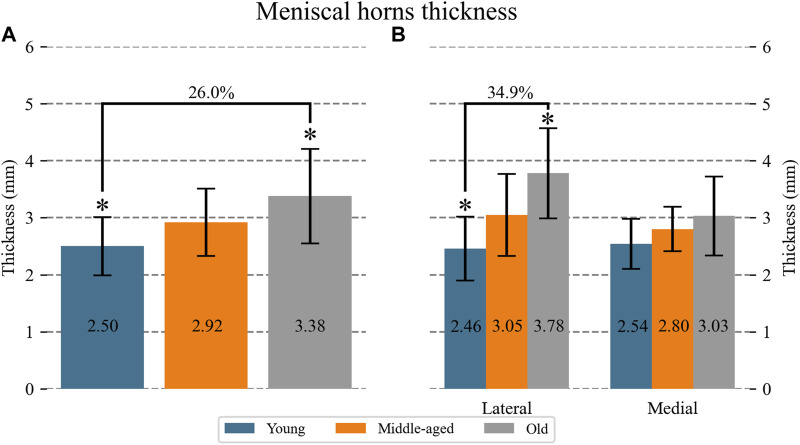
Mean (numerical value in each column) and SD of the thickness of the meniscal horns at the suture hole area: **(A)** for each age group; **(B)** for each age group at the lateral and medial location. For the groups with significant differences, the percentage difference between means with respect to the oldest group is indicated. Significant difference: *Young vs. Old.

An overall difference of meniscus thickness at the suture area was found (*p* < 0.001) (Figure 4A). Pairwise comparisons between the groups showed that the young specimens were 26.0% thinner than the old ones (*p* < 0.001), and a tendency to significance was found between the middle-aged and the old group with the middle-aged menisci being 13.6% thinner. No significant differences were found between the young and middle-aged groups.

Figure 4B shows meniscus thickness differentiating the lateral from the medial location. An overall difference of meniscus thickness at the suture area was found for the lateral meniscus (*p* = 0.005). Pairwise comparisons showed that old lateral specimens were 34.9% thicker than young lateral ones (*p* < 0.001). No differences were found for the medial menisci.

### 3.2 Meniscal cut-out force, Fc

**FIGURE 5:**
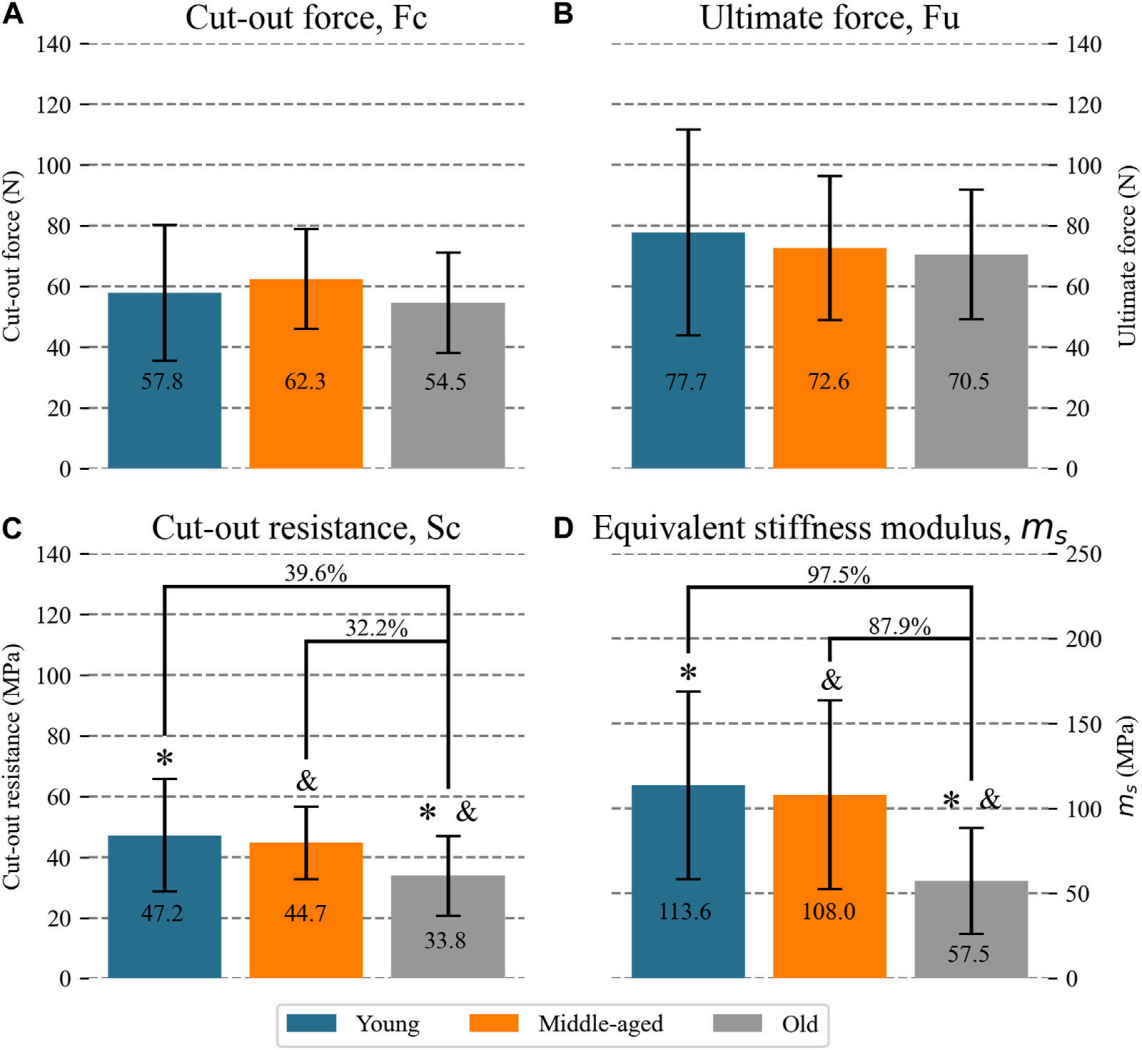
Mean (numerical value in each column) and SD of mechanical properties of the meniscal horns for each age group: **(A)** cut-out force **(B)** ultimate force **(C)** cut-out resistance **(D)** equivalent stiffness modulus. For the groups with significant differences, the percentage difference between means with respect to the oldest group is indicated. Significant difference: *Young vs. Old; ^&^ Middle-aged vs. Old.

At the specimen level, the resistance to suture traction, $F_{c}$ , is shown in Figure 5A for all groups. No significant differences were found between human meniscal horns of different ages, with means differing by only 7.2% between the young and middle-aged groups, 6.1% between the young and old groups, and 14.4% between the middle-aged and old groups.

**FIGURE 6:**
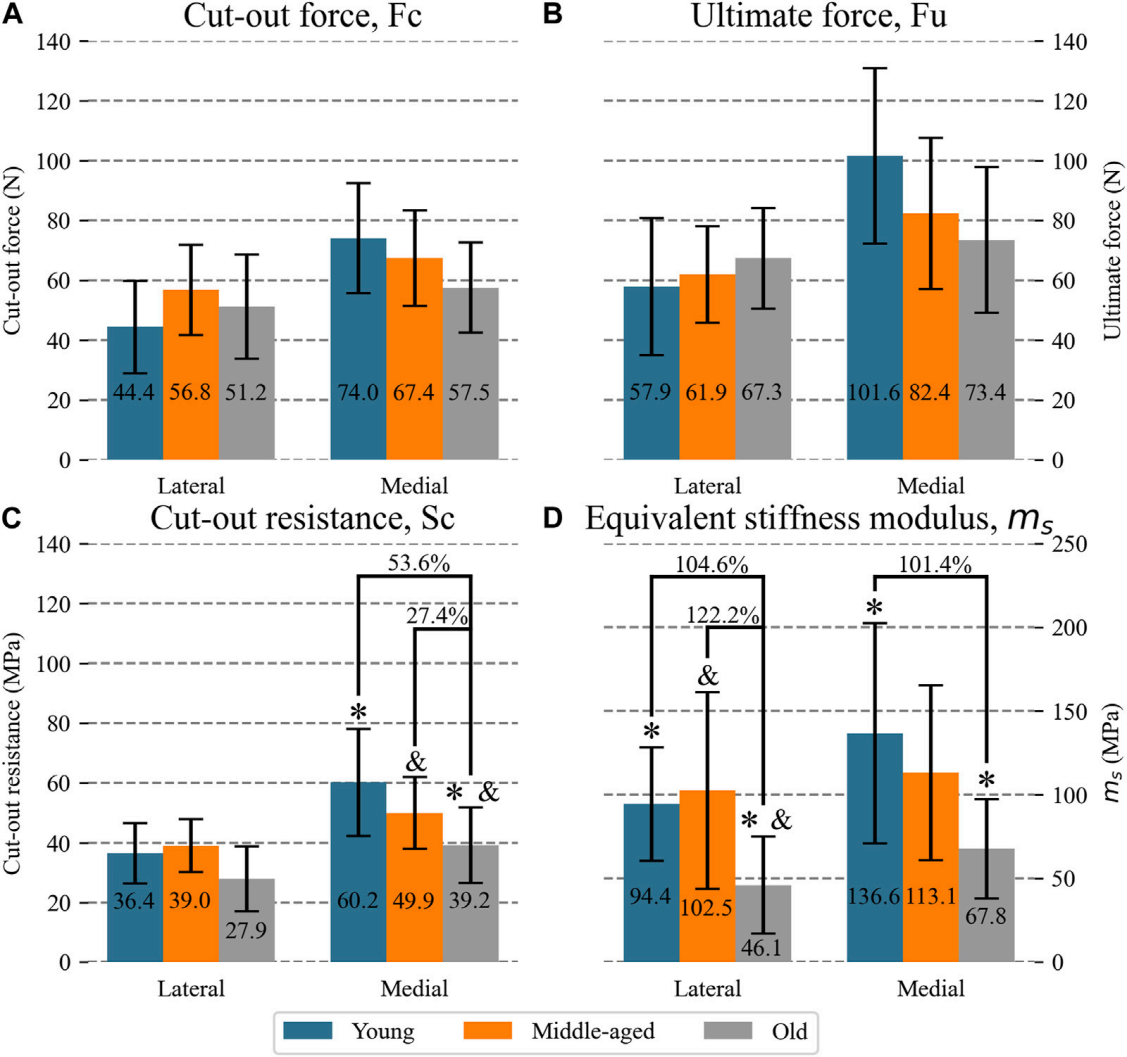
Mean (numeric value in each column) and SD of mechanical properties of the lateral and medial meniscal horns for each age group: **(A)** cut-out force; **(B)** ultimate force **(C)** cut-out resistance **(D)** equivalent stiffness modulus. For the groups with significant differences, the percentage difference between means with respect to the oldest group is indicated. Significant difference: *Young vs. Old; ^&^ Middle-aged vs. Old.

Figure 6A presents mean and SD values of $F_{c}$ differentiating between the lateral and medial meniscus within each human group. No differences were found between the age groups at any location.

### 3.3 Meniscal ultimate force, $F_{u}$

Ultimate load (Figure 5B) was similar in the human groups of different ages, with means differing by only 7.1% between the young and middle-aged groups, 10.3% between the young and old groups and 2.9% between the middle-aged and old groups. The mean value of $F_{u}$ was 26.4% higher than $F_{c}$ for the tested menisci.

When distinguishing between anatomical sites (Figure 6B), no differences between age groups could be found either.

### 3.4 Tissue cut-out resistance, Sc

Figure 5C shows the mean and SD of the tissue cut-out resistance, $S_{c}$ , for all the groups. An overall difference was found between the groups (*p* = 0.012). The sutured tissue was less resistant in the older group than in the young group with a mean S_c_ which was 39.6% higher in the latter (*p* = 0.015) and showed a tendency to significant differences between the middle-aged and old groups, the mean S_c_ being 32.2% higher in the middle-aged group. No differences were found between the young and middle-aged groups.

When comparisons were carried out between the horns proceeding from the lateral meniscus (Figure 6C), no overall difference was found between the groups. However, for the horns proceeding from the medial meniscus, after having detected an overall difference (*p* = 0.008), the tissue from the old group was less resistant than the other two groups (*p* = 0.03 young vs. old; *p* = 0.03 middle-aged vs. old) by 53.6% with respect to the young group and 27.4% with respect to the middle-aged group.

### 3.5 Equivalent stiffness modulus, m_s_

All meniscal horns showed highly linear behavior in the strain range $\left[0-0.3\right]$ , where the equivalent stiffness modulus of tissue at the sutured area, m_s_, was computed. The adjusted R-square of the linear fit always resulted greater than 0.91.

Concerning the influence of age in human specimen behavior, an overall difference between the groups was found (*p* < 0.001). Meniscal tissue at the sutured area resulted more elastic in the old group than in the young and middle-aged groups (*p* < 0.002 young vs. old group; *p* = 0.004 middle-aged vs. old group), m_s_ being 97.5% higher in the young group and 87.9% higher in the middle-aged group than in the old group (Figure 5D). No significant differences were found between the young and middle-aged groups.

Similar results were found when the influence of age was analyzed differentiating between the lateral and medial meniscus (Figure 6D). For the lateral menisci (overall difference *p* = 0.005), the old group showed an m_s_ which was 104.6% lower than the young group (*p* = 0.009) and 122.2% lower than the middle-aged group (*p* = 0.012). No differences were found between the young and middle-aged groups. At the medial location (overall difference *p* = 0.008), the old group showed an m_s_ which was 101.4% lower than the young group (*p* = 0.012) and 66.8% lower than the middle-aged group, with a tendency to significance. No significant differences were found between the young and middle-aged groups either.

## 4 Discussion

This paper studies the influence of age on the mechanical behavior of the sutured meniscal horn in the immediate postoperative period, distinguishing between young (less than 55 years old), middle-aged (between 55 and 75 years old) and old (older than 75 years old) specimens. The results of *in vitro* experimentation revealed that, at the suture hole area, meniscal specimens older than 75 years were more elastic and had a tissue which was less resistant to cut-out. However, a meniscal thickening with age was also found, which leveled out the differences in the maximum force that the horn could withstand by suture traction.

Regarding the deformation of the meniscal tissue at the surroundings of the suture hole prior to cut-out initiation, it was verified that in the strain interval $\left[0-0.3\right]$ it was well represented by the slope of a linear model, i.e., the equivalent stiffness modulus. In terms of this parameter, old meniscal tissue was considerably more elastic than young tissue. The difference was clear between the group of over 75 years old and the two younger ones, with a greater difference in the mean elasticity between the two groups most distant in age (87.9% and 97.5%, respectively). However, no differences were found between the two groups below 75 years of age. Regarding the ability of the meniscal horn to withstand suture traction, it was found that the tissue cut-out resistance, measured as the stress required to initiate tearing, decreased with age and again showed a clear difference between the older group and the two younger ones. However, this deterioration of tissue was not reflected in a decrease of the cut-out resistance of the human meniscal horn, i.e., the force required to initiate cutting, or the maximum force borne by the tissue in the load-to-failure test, since no differences were found between the age groups for these variables. The thickening of the meniscal horns with age may explain the absence of differences in the maximum forces prior to tissue cutting despite the weakness of the tissue, since such thickening increases the resistance area (denominator in equation 3), i.e., although the meniscal tissue becomes clearly less resistant with age (Figure 5C) no difference could be detected for the maximum pulling force previous to tearing (Figure 5A) or ultimate force (Figure 5B).

When the effect is disaggregated between the lateral and medial meniscus, the analysis results less conclusive, likely due to the lower statistical power of these comparisons, since the sample size was computed for the groups without distinguishing between anatomical sites. For the lateral menisci, no influence of age could be detected in either tissue resistance or in the ultimate force or cut-out force, but a thickening of the old group compared to the young group was observed. For the medial menisci, no influence of age on its thickening was detected, but a decrease in tissue cut-out resistance for the old group with respect to the two younger groups was observed with no detectable differences in the meniscal cut-out force or ultimate force. With regards to the deformation in the suture zone, a greater deformation was detected in the older age group in both anatomical sites, consistent with the findings of the global study.

The observed changes in biomechanical properties with age could have multifactorial causes, probably due to histologic or biochemical alterations in the tissues. This study represents a first step towards comprehending the impact of age on the performance of the sutured meniscus. Once significant changes in resistance and elasticity have been confirmed, further investigation into the underlying causes of such variations would aid in comprehending this occurrence.

In biomechanical researches, sutured meniscal models are commonly used to *in vitro* evaluate surgical repair techniques and devices. Both the resistance of the repair to withstand loads that induce traction on the suture (Anz et al., 2014; Mitchell et al., 2016) and post-repair displacements generated by such loads (Vertullo et al., 2021) are computed to check if they remain within the clinically admissible limits or which option most closely approximates natural biomechanics. In light of the results of the study, the authors recommend restricting the age of donors to under 75 years for *in vitro* tests involving sutured meniscal roots whose results are to be applied to patients below 75 years of age. Similarly, if the results are to be applied to patients above 75 years, the menisci should be obtained from donors older than 75 years of age.

For menisci older than 75 years old, the displacements prior to the possible initiation of a tear would be greater, which may compromise the success of the intervention. Indeed, it has been reported that after meniscal root reinsertion, displacements greater than 5 mm can alter the contact biomechanics of the repaired knee (Sekaran et al., 2002; Prado-Novoa et al., 2020). In contrast, we could not verify a decrease in the traction resistance of the sutured horn of over 75 years of age. The weakening of the tissue above this age is counteracted by thickening of the meniscal horn. This shift in meniscal anatomy was observed in a previous study (Seitz et al., 2021), although the authors distinguished between mildly and severely degenerated menisci groups, also resulting differentiated by the age of the donor.

From a clinical point of view, the authors find no reason not to extend meniscal repairs with sutures to patients up to 75 years of age. This reasoning excludes biological considerations that might influence improved healing in younger patients, and other clinical factors that cannot be addressed in this *in vitro* testing.

Whether the mechanical properties of one of the meniscal sites, lateral or medial, are more affected by ageing has also been evaluated in this research. Regarding the deformation at the sutured area, a similar influence of age was found at both compartments, i.e., greater elasticity of the older group. The percentage differences between group means were very high for this variable. A decrease in resistance was also observed at the tissue level, but it was only significant in the medial meniscus. No other differences were found, probably because the study design was underpowered for this analysis, preventing us from drawing any conclusions. In light of this results, we believe that further research on the influence of age on each specific root would be beneficial.

To our knowledge, this is the first research to deal with the influence of age on the biomechanical response of the meniscal tissue to a suture. The cut-out resistance of the meniscal horns to the traction from the suture has previously been assessed with concordant results to ours, although the authors focused on evaluating different suturing techniques or devices for root reattachment and always disregarded the influence of aging. Antz et al. (Anz et al., 2014) tested human menisci, aged 46–64 years, in a load-to-failure test by pulling from the posterior horn sutured with two simple stitches with a N°2 surgical suture of UHMWPE. The initial peak on the load-displacement curve was computed, considering it the starting point of loss of structural integrity of the sample, i.e., the cut-out initiation point. Despite differences in testing protocols, the reported values are in line with our results for the middle-aged group: a mean initial peak force of 137 ± 49 N with two stitches which approximately double the 62.3 ± 16 N that we found for F_c_ with a single stitch. Vertullo et al. (Vertullo et al., 2021) reported an ultimate load of 94.29 ± 7.99 N for a group of posterior medial menisci with a mean age of 54 ± 4-year-old (no age range reported) with two simple No. 2 sutures, which is also compatible with our results, taking into account that the samples were previously weakened by 1,000 cycles of load between 5 N and 20 N and two stitches were used. Asymmetries in the traction distribution between the 2 stitches frequently occur making it difficult to predict the ultimate load of the construct. Mitchell et al. (Mitchell et al., 2016) found a mean ultimate force of 58.2 ± 29.6 N, when testing in posterior horns of human medial menisci in the range 48–88 years and sutured with one simple stitch. The values reported are clearly lower than our results for $F_{u}$ , likely because the authors used a Nº0 surgical suture of UHMWPE which implies a smaller diameter, and consequently a smaller resistant area, a 42.9% thinner than in our work.

Regarding meniscal horn elasticity, the accumulation of advanced glycation end-products (AGEs) have been related to an increase in stiffness of soft tissues other than the meniscus, like tendons. As AGEs accumulation has been observed in menisci with age, some researchers have hypothesized that menisci would behave in the same way and therefore their stiffness should increase with aging (Tsujii et al., 2017; Sarbacher and Halper, 2019). However, our results contradict this hypothesis. We are not aware of any studies that calculates the equivalent stiffness modulus of the sutured meniscus tissue subjected to traction from the suture. We only know of biomechanical works that report the value of the stiffness of the suture-meniscus construct. Furthermore, these results are widely dispersed even for the same repair technique (Mitchell et al., 2016; Vertullo et al., 2021), making comparison with our outcomes difficult. The dispersion in these works is affected by factors unrelated to meniscal tissue, which is not involved in our results, such as free lengths of suture threads, which is not always informed, or histories of cyclic loading prior to load-to-failure test, which are known to influence on the elasticity of these surgical sutures if the resting time is not long enough (Prado-Novoa et al., 2022).

In our *in vitro* study, in accordance with other researches (Zantop et al., 2004; Feucht et al., 2013; Anz et al., 2014; Cerminara et al., 2014; Mitchell et al., 2016; Perez-Blanca et al., 2018; Prado-Novoa et al., 2020; Vertullo et al., 2021), we opted to use manually operated mechanical grippers to attach the meniscus and suture to the testing machine, instead of the also used pneumatic clamps (Massey et al., 2019). This choice provides gradual and soft control over the grasping forces, especially for the meniscus, allowing careful management of potential damage to the specimen at the clamping area. To validate the grasping effectiveness and discard possible slip-induced errors, lines were marked on the meniscus and the suture at their respective boundaries with the jaw and clamp. These markings were then monitored using the videogrammetric system, as detailed in the “Methods” section.

Limitations inherent to *in vitro* studies are relevant to this work, such as the absence of the aforementioned healing effect over time. Also, although the main clinical application of this research is the evaluation of the influence of age on the viability of meniscal root repairs, as well as the suitability of different age models for the *in vitro* study of such operations, isolated meniscus-suture constructs were tested instead of attaching menisci to tibial bones. Therefore, the effect of the surrounding soft tissue, ligaments, cartilage and bones, key players in biomechanical performance of the meniscus, was not included. This setup was chosen as in other works (Anz et al., 2014; Mitchell et al., 2016; Perez-Blanca et al., 2018; Vertullo et al., 2021) to focus on the behavior of the tissue-suture interface, disregarding other factors that depend on tibial fixation. For the same reason, as in previous studies (Anz et al., 2014; Mitchell et al., 2016; Perez-Blanca et al., 2018; Vertullo et al., 2021), the suture was aligned with the loading direction, considering that pulling in the direction of the suture eliminated shear friction and focused the tensile effect on the meniscus-suture interface which was our main interest. The meniscal horn fibers were also aligned with the loading direction to maximize damage to the tissue, a conservative criterion to detect the maximum load that the suture repair would withstand. Alignment of the traction load with the suture and with the horn fibers did not reproduce any particular anatomical situation but focused the tensile effect on the meniscus-suture interface, in keeping with the main interest of our study. A single simple suture was performed at the meniscal horn, despite this not being the most common surgical option (Anz et al., 2014; Mitchell et al., 2016; Perez-Blanca et al., 2018; Vertullo et al., 2021). The use of a single suture allows a better computation of the forces that cause the tear. When multiple stitches are performed the pulling load is distributed among them, making it challenging to achieve a uniform distribution between sutures, even under laboratory conditions. Additionally, the use of a simple stitch, although it is neither the most resistant nor the most common surgical solution for meniscal root repair, facilitates the quantification of the meniscus-thread contact area, which is required for calculating the stress at the suture-meniscus interface, as it is less affected by the meniscus anatomy or the surgeon’s performance compared to more complex solutions such as the loop stitch, Kessler stitch, locking stitch, etc., or reinforced suture techniques, as those proposed for meniscal tears (Massey et al., 2020). The load-to-failure test was performed at 0.1 mm/s, a representative velocity of a quasi-static displacement and lower than expected for knee movement in daily activity (Prado-Novoa et al., 2022). This minimizes the influence of possible viscous effects on the test, a characteristic that is not dealt with in this study. However, given the viscoelastic behavior of the meniscus reported in compression tests (Norberg et al., 2021; De Rosa et al., 2022; Morejon et al., 2022), further research is warranted to comprehensively understand the dynamic response of the sutured meniscal tissue. The cut-out initiation was detected by using two methodologies to ensure its accurate location. Initially, it was detected automatically with the method validated by Perez-Blanca et al. (Pérez de la Blanca, 2019) and subsequently the accuracy of the identification was verified in video images. Our results showed that, as previously reported (Pérez de la Blanca, 2019), the tearing initiated at a load which was very close to but slightly before the first local maximum of the load-deformation curve.

## 5 Conclusion

Regarding the influence of age on sutured meniscal horn tissue, *in vitro* experimentation revealed that meniscal horn specimens older than 75 years had a tissue at the suture hole area that was more elastic and less resistant to cut-out than younger menisci. However, a thickening of the meniscal horns with age also occurs, which leveled out the differences in the force that initiates the tear, as well as in the maximum force borne by the meniscus in a load-to-failure test.

## Data Availability

The raw data supporting the conclusion of this article will be made available by the authors, without undue reservation.
