# Supplementary material for: Age influence on resistance and deformation of the human sutured meniscal horn in the immediate postoperative period
Source: Front Bioeng Biotechnol. 2024 Jan 5;11:1249982. doi: 10.3389/fbioe.2023.1249982 (PMC10796521; doi:10.3389/fbioe.2023.1249982)
Supplement: Supplementary file 1 [file DataSheet2.pdf]

Supplementary Material

Age influence on resistance and deformation of the human sutured meniscal horn in the immediate postoperative period

Alejandro Peña-Trabalon*, Ana Perez-Blanca, Salvador Moreno-Vegas, M. Belen Estebanez Campos, Maria Prado-Novoa

*** Correspondence:** alejandropt98@uma.es

The videogrammetric software, implemented in MATLAB®, allows the identification of the two marks on the meniscus surface. It enables the extraction of their coordinates in each calibrated frame, subsequently computing the distance between them. The software processes all the frames captured during the load-to-failure test.

The comprehensive algorithm comprises the following sections.

a) Calibration

1. Before the test start, the user is required to capture a calibration image. The calibration image must contain a measurement pattern positioned within the same image plane occupied by the marks on the meniscus surface during the test.

b) Image processing

2. The user specifies the number of marks to be tracked, 2 marks for this application.

3. The user specifies the approximate diameter of the marks in pixels. At this point, the software facilitates the user in measuring this diameter over the initial frame using the MATLAB® ruler.

4. The user selects the colour threshold with a HSV colour model in the initial frame. Subsequently, the software executes a HSV threshold with a hue range between 0 and 0.20, a saturation range between 0 and 0.60, and a value range between 0 and 0.05, all normalized to a maximum of 1. A binarization process with 8 bits is performed on the frame, assigning a value of 0 to pixels that do not meet the threshold and a value between 1 and 255 as the maximum for those that do. Following the binarization, a morphological closing operation is conducted using a disk-shaped structuring element with a radius of 4 pixels to smooth borders and fill any holes in the identified regions. Additionally, a minimum area filter of 50 pixels^2 is applied to exclude small isolated pixels that are not associated with any mark. To further refine the results, an intensity grayscale threshold of 50 is applied, eliminating areas that meet the colour threshold but have lower intensity than specified. Only areas with a pixel value exceeding 50 are considered suitable and retained in the frame.

5. Point 4 is applied uniformly across all frames acquired by the cameras during the load-to-failure test.

c) Calculation of coordinates of each mark

6. In the first frame, the user identifies the starting position of each mark to be tracked.

7. Using the position of each mark in the first frame as the initial reference, the algorithm confines its search for circular regions corresponding to the marks in the subsequent frame within a 10x10 pixel area around them. Consequently, each frame employs the coordinates identified in the preceding frame, streamlining the identification process, and mitigating high time consumption. This iterative procedure is applied to all captured images.

8. A list containing the 2D coordinates of the centroid of the marks in all the frames is generated with the coordinates expressed in pixels.

9. Using the calibration image of point 1, the coordinates are converted from pixels to millimeters.

d) Calculation of the distance between marks

10. The distances in millimeters between marks are calculated across all the frames, thereby providing the evolution of the displacement between them throughout the load-to-failure test.

11. Values are saved into a MATLAB® file.
