# Supplementary material for: Age influence on resistance and deformation of the human sutured meniscal horn in the immediate postoperative period
Source: Front Bioeng Biotechnol. 2024 Jan 5;11:1249982. doi: 10.3389/fbioe.2023.1249982 (PMC10796521; doi:10.3389/fbioe.2023.1249982)
Supplement: Supplementary file 2 [file DataSheet1.pdf]

frontiers

Supplementary Material

Age influence on resistance and deformation of the human sutured meniscal horn in the immediate postoperative period

Alejandro Peña-Trabalon*, Ana Perez-Blanca, Salvador Moreno-Vegas, M. Belen Estebanez Campos, Maria Prado-Novoa

*** Correspondence:** alejandropt98@uma.es

1 Supplementary Data

Each age group was divided into specimens tested on either the anterior or posterior horn. The young group resulted in 2 groups of N=11 specimens (for anterior horn group: 7 medial and 4 lateral meniscus, 9 men and 2 women, age 38.90 years, SD 6.15, median 41 years, range 28-47 years; for posterior horn group: 4 medial and 7 lateral meniscus, 9 men and 2 women, age 36.6 years, SD 6.15, median 37 years, range 28-47 years), the middle-aged group resulted in 2 groups of N=11 specimens (for anterior horn group: 5 medial and 6 lateral meniscus, 4 men and 7 women, age 62.82 years, SD 4.42, median 64 years, range 57-67 years; for posterior horn group: 6 medial and 5 lateral meniscus, 4 men and 7 women, age 63.09 years, SD 3.96, median 64 years, range 57-67 years), and the old group resulted in a group of N=10 specimens for the anterior horn (5 medial and 5 lateral meniscus, 6 men and 4 women, age 84.50 years, SD 3.74, median 83 years, range 82-91 years) and a group of N=12 specimens for the posterior root (6 medial and 6 lateral meniscus, 4 men and 8 women, age 85.08 years, SD 4.19, median 83 years, range 82-95 years).

The posterior horns showed differences in thickness between the young and old groups (p=0.03), with the old group being 24.2% thicker than the young group. No other differences were found in the thickness of the specimens.

Regarding the mechanical properties, differences were observed only for tissue-level variables. Specifically, the cut-out resistance of the middle-aged group was 28% higher than that of the old group in the anterior horn (p=0.006). For the posterior horn, the equivalent stiffness modulus was 105.9% higher for the young group (p=0.006) and 118.9% higher for the middle-aged group (p=0.006) both with respect to the old group. No differences were detected between the young and middle-aged groups.

No conclusive findings were reached in the study regarding the impact of age on mechanical properties in either the anterior or posterior horn. For the posterior horn, no differences could be detected in variables regarding resistances. Therefore, the analysis explaining a possible influence of the observed meniscus thickening with age cannot be performed. Regarding the elasticity at the suture area, the behavior was consistent with the findings of the global study, i.e., an increase of elasticity with age. For the anterior horn, only tissue-level resistance differences were found between the middle-aged and old groups, which also does not allow any definitive conclusions to be drawn. Moreover, there was no detectable effect of the age on the elasticity of the sutured tissue in this group.

The lack of conclusion discussed in the previous paragraph is probably due to the small sample size of the groups. Additionally, when distinguishing between the anterior and posterior horn, the design of the study did not ensure equal sample sizes in both locations.

2 Supplementary Figures

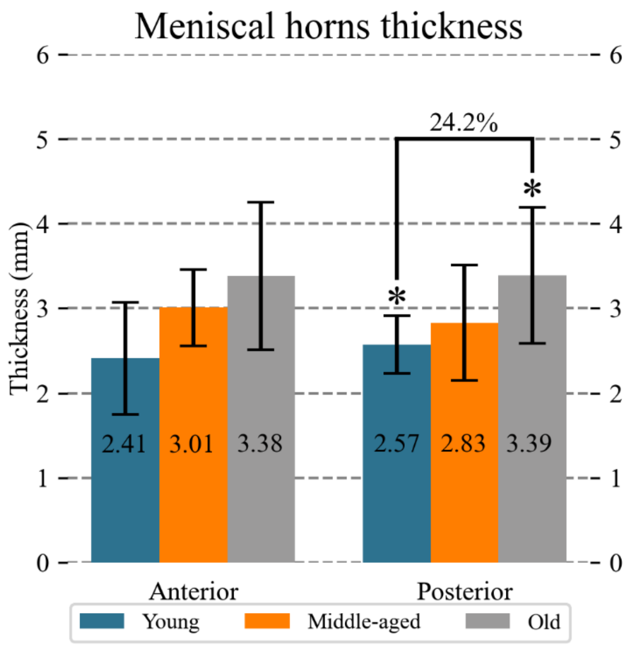

Supplementary Figure 1. Mean (numerical value in each column) and SD of the thickness of the meniscal horns at the suture hole area at the anterior and posterior location for each age group. For the groups with significant differences, the percentage difference between means with respect to the oldest group is indicated. Significant difference: *Young vs. Old.

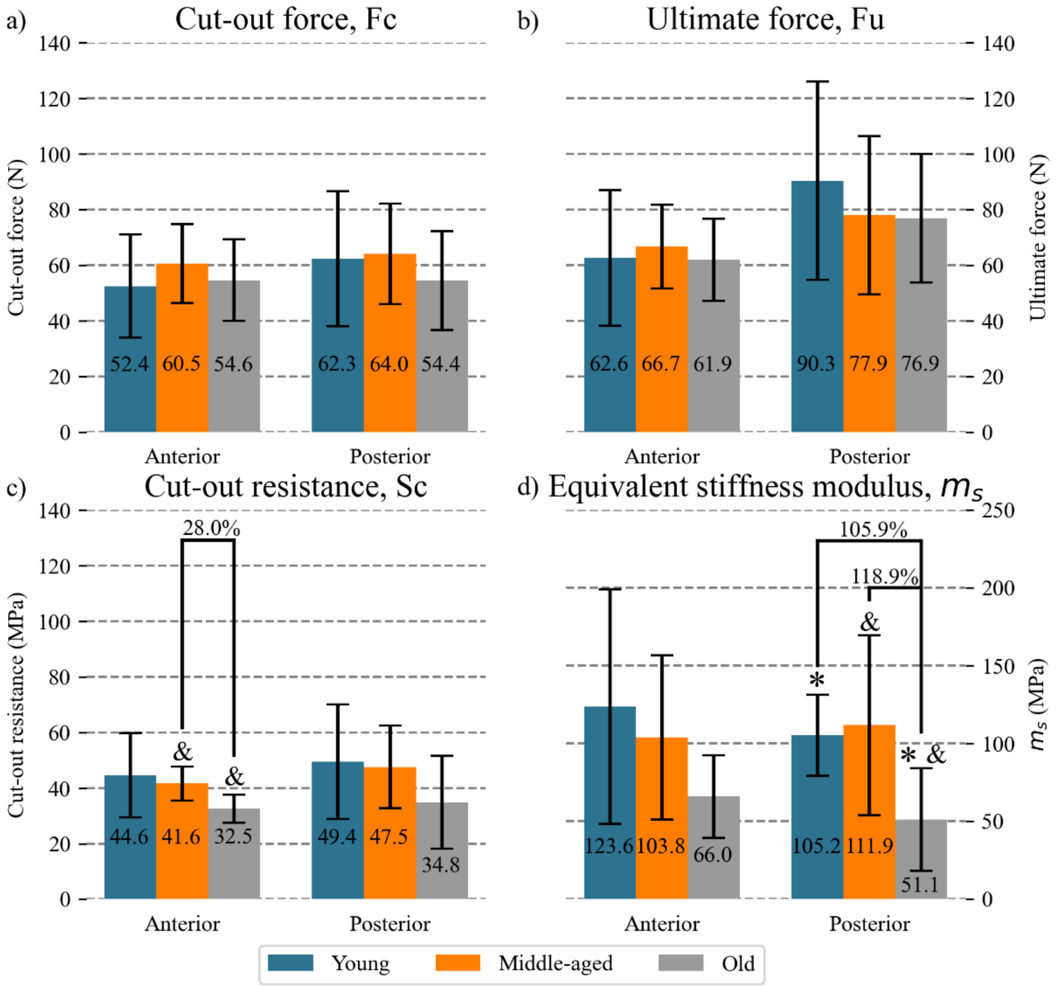

Supplementary Figure 2. Mean (numeric value in each column) and SD of mechanical properties at the anterior and posterior location for each age group: a) cut-out force; b) ultimate force c) cut-out resistance d) equivalent stiffness modulus. For the groups with significant differences, the percentage difference between means with respect to the oldest group is indicated. Significant difference: *Young vs. Old; & Middle-aged vs. Old.
